# EXPLANA: a user-friendly workflow for EXPLoratory ANAlysis and feature selection in cross-sectional and longitudinal microbiome studies

**DOI:** 10.1093/bioinformatics/btaf658

**Published:** 2025-12-19

**Authors:** Jennifer Fouquier, Maggie Stanislawski, John O’Connor, Ashley Scadden, Catherine Lozupone

**Affiliations:** Department of Biomedical Informatics, School of Medicine, University of Colorado, Anschutz Medical Campus, 1890 N. Revere Court, Aurora, Colorado, 80045, United States; Department of Biomedical Informatics, School of Medicine, University of Colorado, Anschutz Medical Campus, 1890 N. Revere Court, Aurora, Colorado, 80045, United States; Department of Biomedical Informatics, School of Medicine, University of Colorado, Anschutz Medical Campus, 1890 N. Revere Court, Aurora, Colorado, 80045, United States; Department of Biomedical Informatics, School of Medicine, University of Colorado, Anschutz Medical Campus, 1890 N. Revere Court, Aurora, Colorado, 80045, United States; Department of Biomedical Informatics, School of Medicine, University of Colorado, Anschutz Medical Campus, 1890 N. Revere Court, Aurora, Colorado, 80045, United States

## Abstract

**Motivation:**

Longitudinal microbiome studies (LMS) are increasingly common but have analytic challenges including nonindependent data requiring mixed-effects models. Furthermore, large amounts of data motivate exploratory analysis to identify factors related to outcome variables. Although change analysis (i.e. calculating feature changes between timepoints) can be powerful, how to best conduct these analyses is often unclear. For example, observational LMS measurements show natural fluctuations, so baseline might not be a reference of primary interest, whereas for interventional LMS, baseline is typically a key reference point, often indicating the start of treatment.

**Results:**

To address these challenges, a feature selection workflow, called EXPLANA (EXPLoratory ANAlysis), was developed for LMS that supports numerical and categorical data, and also accommodates cross-sectional studies. Machine learning methods were combined with different types of change calculations and downstream interpretation methods to identify statistically meaningful variables and explain their relationship to outcomes. EXPLANA generates an interactive report that textually and graphically summarizes methods and results. EXPLANA had good performance on simulated longitudinal data, with a balanced accuracy score of 0.91 (range: 0.79–1.00, SD = 0.05), outperformed an existing tool, QIIME 2 feature-volatility (balanced accuracy: 0.95 versus 0.56) and identified novel order-dependent categorical feature changes (e.g. different effect for A_B versus B_A). EXPLANA is broadly applicable and simplifies analytics for identifying features related to outcomes of interest.

**Availability and implementation:**

Software is available at https://github.com/JTFouquier/explana and https://zenodo.org/records/17478745 (10.5281/zenodo.17478744). Documentation and demos are available at www.explana.io.

## 1 Introduction

Scientific studies often include a collection of complex multiomic data (Santiago-Rodriguez and Hollister 2021), such as microbiome (Ursell *et al.* 2012), transcriptome (Hrdlickova *et al.* 2017), and metabolome (Zamboni *et al.* 2015), and it is of interest to explore whether any novel features, or collections of features, may be related to an outcome variable. Adding to the complexity, researchers often collect other data from individuals that may impact an outcome, such as demographic and health data, or surveys on diet or medications. The growing quantity of available data complicates statistical decisions regarding variable inclusion, which is often based on hypotheses that motivated initial study design. Additionally, studies can include both categorical and numerical variables and can often contain nonindependent longitudinal data, posing greater statistical challenges. As research advancements are made, collaborative efforts with different research laboratories produce more data per study, and human biases are often introduced during study design and analytics. These challenges have ultimately stimulated a growing interest in data-driven methods.

One field particularly impacted by an abundance of data is microbiome research, which focuses on characterizing the community of viruses, fungi, and bacteria and their genes. Characterization of the microbiome is often performed by 16S ribosomal RNA (rRNA) gene sequencing, which identifies the bacteria and archaea in an environment. One well-studied microbial environment is the gut microbiome because of the metabolic potential of the bacterial community and its association with numerous human diseases, including obesity (Maruvada *et al.* 2017), depression (Valles-Colomer *et al.* 2019), autism spectrum disorder (ASD) (Krajmalnik-Brown *et al.* 2015), cancer (Zhuang *et al.* 2019, Rebersek 2021), HIV (Williams *et al.* 2016), and cardiovascular disease (Witkowski *et al.* 2020). The gut microbiome relationship to human disease suggests that gut microbiome modification through interventions like dietary changes, probiotics, or fecal microbial transplants may provide disease prevention or treatment options.

To understand changes in health outcomes and to address the impact of individual variation, longitudinal studies that collect data from multiple individuals, at different timepoints, are essential. In addition to these studies often containing diverse subject data (with numerical and categorical variables), they include repeated measurements on individuals which requires special statistical consideration to identify relationships between features within nonindependent data (Pinheiro and Bates 2000). Random forest- (RF) (Breiman 2001) based machine learning (ML) approaches are powerful for combining different data types to predict outcomes and identify important features. RFs work well with high-dimensional data (more features than samples/instances) (Díaz-Uriarte and Alvarez de Andrés 2006), find linear and nonlinear relationships, and work with nonnormal data distributions. Additionally, RFs are more interpretable than many other ML models because they are based on simple decision trees, which can improve accessibility of complex tools. Additionally, mixed-effects RF (MERF) (Hajjem *et al.* 2014) models can be used for longitudinal study designs. However, numerous challenges can hinder effective application of these methods.

MERFs can be run on original (raw) data from longitudinal studies or by using deltas/changes (Δs) between different reference timepoints, which can reveal unique insights in some studies (Bokulich *et al.* 2018, Ferrocino *et al.* 2018, Meslier *et al.* 2020, Frey *et al.* 2022, Rodenes-Gavidia *et al.* 2023). However, the research question of interest can affect decisions regarding optimal calculation of Δs. In some designs, such as interventions, or some observational studies with an expected trend over time [e.g. gut microbiome changes over the first years of a baby’s life (Bokulich *et al.* 2018)], changes are expected to be compared to a baseline value, so Δs can be calculated using baseline as a reference (Ferrocino *et al.* 2018, Frey *et al.* 2022). However, some observational studies have no meaningful baseline, and it might be of interest to relate an outcome variable to changes in predictors between adjacent timepoints or all pairs of timepoints (Fouquier *et al.* 2021, Zhang *et al.* 2021). For instance, in an observational longitudinal study of children with ASD that we conducted (Fouquier *et al.* 2021), children with ASD were evaluated over time to identify relationships between ASD-associated behaviors and diet, gastrointestinal distress, and the microbiome. Because of high interpersonal gut microbiome variation, this LMS revealed relationships between the gut microbiome and ASD behaviors as a correlation between the degree of microbiome change and ASD behavior change between timepoints. However, because more than two timepoints were studied, and because the first timepoint was not a meaningful baseline, pairwise analyses were performed. Pairwise analysis is useful for identification of effects that are time delayed (e.g. a change from time 2 to time 4), order-dependent, or reference-dependent. Different longitudinal study designs highlight the importance of understanding changes in features, for each subject over time, and that feature changes differ depending on their reference values. Statistical methods differ regarding how and when to apply change analysis and can even lead to different conclusions (Twisk 2013). Thus, methods are needed to compare results from original and Δ datasets for a more complete picture of a longitudinal study.

**Figure 1.**
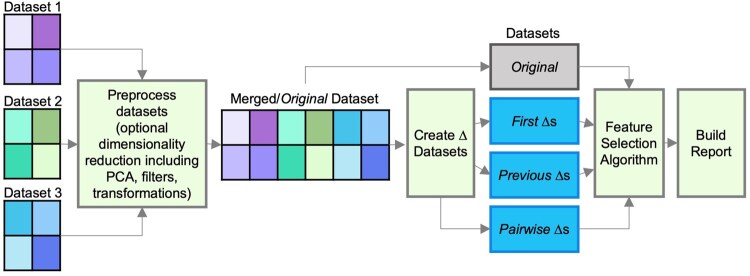
Feature selection workflow diagram. Individual datasets can be preprocessed separately to reduce dimensionality using a variety of methods, including PCA, CLR transformation, or filters. Datasets are merged to form the *Original* dataset prior to creation of Δ datasets for longitudinal studies. *First*, *Previous*, and *Pairwise* Δ datasets are created as explained in Fig. 2. During Δ dataset creation, distance matrices can be incorporated. Feature selection is performed for up to four models built from each dataset (*Original*, *First*, *Previous*, and *Pairwise*). An .html report is created which summarizes features selected per model.

Another analytic challenge encountered in the application of RFs to complex microbiome studies is the integration of microbiome data as predictor variables (i.e. features) with other data types (e.g. surveys or clinical reports with numerical and categorical data). To the best of our knowledge, there are no software tools that create and select order-dependent categorical feature changes that impact an outcome variable. For example, the drugs amiodarone and quinidine for heart arrhythmia treatment have an interaction that could lead to a dangerously rapid heartbeat (Tartini *et al.* 1982), but an interaction risk is higher if amiodarone precedes quinidine since amiodarone has a much longer elimination half-life [days (Vassallo and Trohman 2007) versus hours (Mullen *et al.* 2018)]. This example highlights how calculating order-dependent categorical Δs might uncover relationships that have differential impact if introduced in opposite order, such as in crossover study designs (AB/BA designs). This led us to the hypothesis that unique features dependent on different contexts of change could be identified, including novel order-dependent categorical features by tracking text changes as an engineered feature value (e.g. “amiodarone__quinidine”; Fig. 1, available as supplementary data at *Bioinformatics* online).

Finally, another key challenge is performing complex LMS analytics in a reproducible way that facilitates communication about results. These workflows can involve inputs of diverse data types, calculation of Δs with different reference points, feature selection using mixed-effects ML methods, and methods for explaining why features were selected, in addition to their importance ranks. Although there are tools for feature selection in microbiome data (Bokulich *et al.* 2018, Lee *et al.* 2020, Queen and Emrich 2021, Wang *et al.* 2021, Bodein *et al.* 2022, Calle *et al.* 2023), none provide the combination of methods described here. For example, timeOmics (Bodein *et al.* 2022) is useful for multiomic integration with an emphasis on time as the outcome, while other goals are to identify features related to different outcome variables over time. QIIME 2 (Q2) (Bolyen *et al.* 2019) longitudinal feature-volatility (FV) (Bokulich *et al.* 2018), a feature selection tool provided as part of a very popular microbiome analytics platform, allows for evaluation of different outcomes, but does not incorporate metrics that explain the selected feature’s impact on an outcome. Both tools, although useful for longitudinal analytics, do not incorporate categorical Δs. Although individual tools for data preprocessing, application of RFs or MERFs, and downstream interpretation of results also exist, it is cumbersome for scientists to research and implement this complex array of tools.

For these reasons, EXPLoratory ANAlysis (EXPLANA) was developed as a data-driven feature selection workflow to streamline hypothesis generation for longitudinal data, while accommodating cross-sectional data and both numerical and categorical variables. EXPLANA can identify unique features important in different contexts of change, including order-dependent categorical features related to changes in outcomes. Results are presented in an interactive report to document complex analytics. The combination of novel and existing methods to address analytic challenges provides broad applicability for scientific discovery.

## 2 Materials and methods

### 2.1 Workflow overview

**Figure 2.**
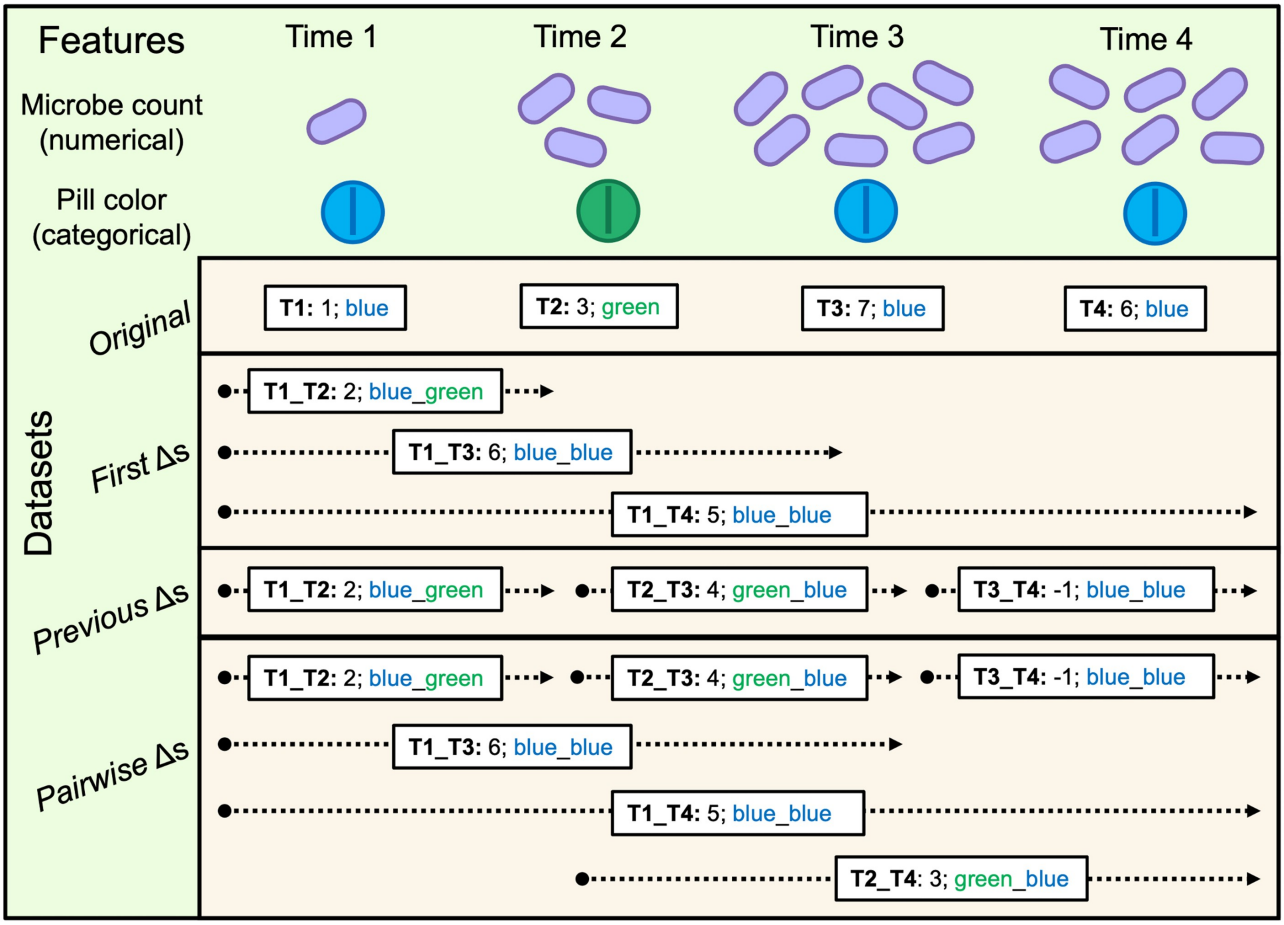
Example calculations of *First*, *Previous*, and *Pairwise* Δ datasets for numerical and categorical features in a four timepoint study. The *Original* dataset includes original feature values (without change analysis) and Δ datasets contain differences/changes in features, per subject, between timepoints. Different reference points are used for each Δ dataset. For categorical features (e.g. pill color), text is used to track the order of categorical changes and for numerical features (e.g. microbe count), the reference is subtracted from the later timepoint. The comparison between two timepoints is indicated before each colon (e.g. a Δ between timepoint 1 and 2 is indicated as T1_T2). For a four timepoint study: *First* Δs: T1_T2, T1_T3, T1_T4; *Previous* Δs: T1_T2, T2_T3, and T3_T4; and *Pairwise* Δs: T1_T2, T2_T3, T3_T4, T1_T3, T1_T4, and T2_T4.

EXPLANA was developed using Snakemake (Köster and Rahmann 2012) to facilitate piping inputs and outputs from scripts written in different software languages, primarily R and Python (Fig. 1). The workflow is executed from user-input arguments from a configuration file which pipes files to different scripts concluding with an .html report. The configuration file includes a list of datasets in tabular format [microbiome (taxa or amplicon sequence variants defined with 16S rRNA, or genes/taxa defined using shotgun metagenomic data) surveys, demographics, metadata, etc.], to be provided in long format (rows are samples; columns are features). For longitudinal data, every row in each dataset must correspond to a unique sample identifier that represents a subject and timepoint (Subject1_time1, Subject1_time2). First, individual datasets can be preprocessed through filters, dimensionality reduction, or transformation. If multiple files exist, they are merged to create the *Original* dataset. For longitudinal data, Δ datasets are computed (Fig. 2). Finally, a feature selection algorithm is implemented by building a model for each of the four datasets (*Original, First, Previous*, and *Pairwise*): First, RFs or MERFs (Hajjem *et al.* 2014) (for multiple samples per subject) are trained; next, BorutaSHAP (Keany 2021; https://pypi.org/project/BorutaShap/) is used to rank features by importance if they perform better than expected by random chance, and determine feature impact on response. The final report includes figures, tables, and a written analytic summary.

Analyses were completed locally to ensure reasonable compute time for typical academic microbiome studies or those without server access. For 1000 features, 5 timepoints and 100 individuals, run time is <30 min using a MacBook Pro (Memory: 32 GB 2400 MHz DDR4; Processor: 2.9 GHz; 6-Core Intel Core i9).

### 2.2 Software and data availability

Workflow documentation and additional demos can be found at www.explana.io and software, implementation, sample datasets, and licensing information at https://github.com/JTFouquier/explana.

### 2.3 Configuration file

Each configuration file is associated with one analysis which is modified to specify datasets, a response/outcome variable, sample identifier column, timepoint column, distance matrices (if applicable), optional dimensionality reduction steps prior to feature selection, and ML method decisions/arguments. Feature values, as well as feature columns, can be kept or dropped for individual datasets or for the merged *Original* dataset using small scripts within the configuration file.

### 2.4 Preprocessing datasets

For each feature selection analysis, one or more dataset files (typically data frames in a tabular format) can be used as needed. For example, dataset 1 may contain microbiome data, dataset 2 may include clinical variables, and dataset 3 may consist of survey responses. Microbiome data can be input as rarefied, or with or without transformation. Each dataset can be preprocessed, which may include dropping features or feature values, on a per dataset basis. Dimensionality reduction can be performed prior to feature selection using principal components analysis (PCA) or filters. Transformation is also possible with arcsin or center-log-ratio (CLR) for microbiome data. Principal coordinates analysis (PCoA) should be used with microbiome datasets for the whole community dimensionality reduction and is commonly part of upstream microbiome platforms. Users may want to generate PCoAs from relevant phylogenetic or nonphylogenetic distance matrices during upstream sequence analysis. For nonmicrobiome variables, PCA can be used on a set of related variables to capture the maximum variance using fewer variables and was included to handle the varied choices with other datasets (e.g. for creating a score from disease symptom surveys or metabolic markers). Short scripts can be added to the configuration file to modify each dataset or the complete dataset after merging individual datasets.

### 2.5 Merging datasets

After preprocessing, individual data frames are merged using a shared sample identifier column to create the “*Original”* dataset with variables from all datasets. The *Original* dataset is named accordingly because it contains original values of features that may have been sampled over time (i.e. *Original* does not include intraindividual changes/differences between timepoints like the Δ datasets). Data merges prioritize samples in the top/first dataset. This means additional samples in other datasets will not be included. For some analyses or complex data frames (e.g. lots of missing data, duplicated columns, etc.), merging data prior to workflow implementation may be simpler and care should be taken to ensure merge accuracy, as this is a basic merge step.

### 2.6 Delta (Δ) dataset creation

For longitudinal analyses, the *Original* dataset is used to compute three Δ datasets, *First*, *Previous*, and *Pairwise* by calculating feature changes over time, per subject, using different reference points (Fig. 2). Δ dataset calculations: For *First*, compared to baseline/first; for *Previous*, compared to previous timepoint; for *Pairwise*, all pairwise comparisons between timepoints. For two timepoint studies, only *Original* and *First* are needed.

For categorical variables, the order of categorical values for each subject at both timepoints per comparison is tracked (e.g. for pill color, if T2 is green and T3 is blue, then T2_T3 is green_blue). For numerical variables, reference values are subtracted from the later timepoint (e.g. if T2 is 3 and T3 is 7, Δ = 4). Timepoint is numerical for *Original* to provide information about order of events, and categorical for Δ datasets due to overlap in timepoints (e.g. T1_T2 and T1_T3 overlap each other at T1_T2). This overlap can be thought of as though time were categorical rather than an abstract concept. In other words, if T1, T2, and T3 were recoded as A, B, and C, respectively, the comparisons A_B, A_C, and B_C are potentially interesting.

### 2.7 Feature selection algorithm

Feature selection is performed using all four models, as needed. For categorical features, unique values/classes per feature are encoded to binary features (labeled as “ENC”), where feature presence in a sample is 1 and absence is 0. This enables selection of uncommon feature values that influence an outcome variable.

Next, RF regression is performed to select features related to the outcome. When more than one measurement per subject exists, MERF is used. Both use Scikit-Learn (Pedregosa *et al.* 2011) RandomForestRegressor as the fixed effects forest. Boruta (Kursa and Rudnicki 2010) is a method that uses shuffled versions of input features to assess whether importance scores are better than random chance. Features are categorized as accepted, tentative or rejected. BorutaSHAP is implemented because it works with the unique properties of SHAP (SHapley Additive exPlanations) (Lundberg and Lee 2017), which provides feature ranks and estimated impact on the outcome.

Rejected features are dropped, RF (or MERF) is rerun, and visualizations are generated without irrelevant features that might hide true signal from important features. Final percent variation explained (*R*^2^) comes from out-of-bag (OOB) scores from the complete-feature forest. For additional context, the percent variation explained by the reduced-feature forest is also provided. OOB scores are used for internal validation and created from leaving out some samples for each decision tree in the forest during training and comparing the tree’s results to the real outcome values for the samples left out.

### 2.8 Report details

**Figure 3.**
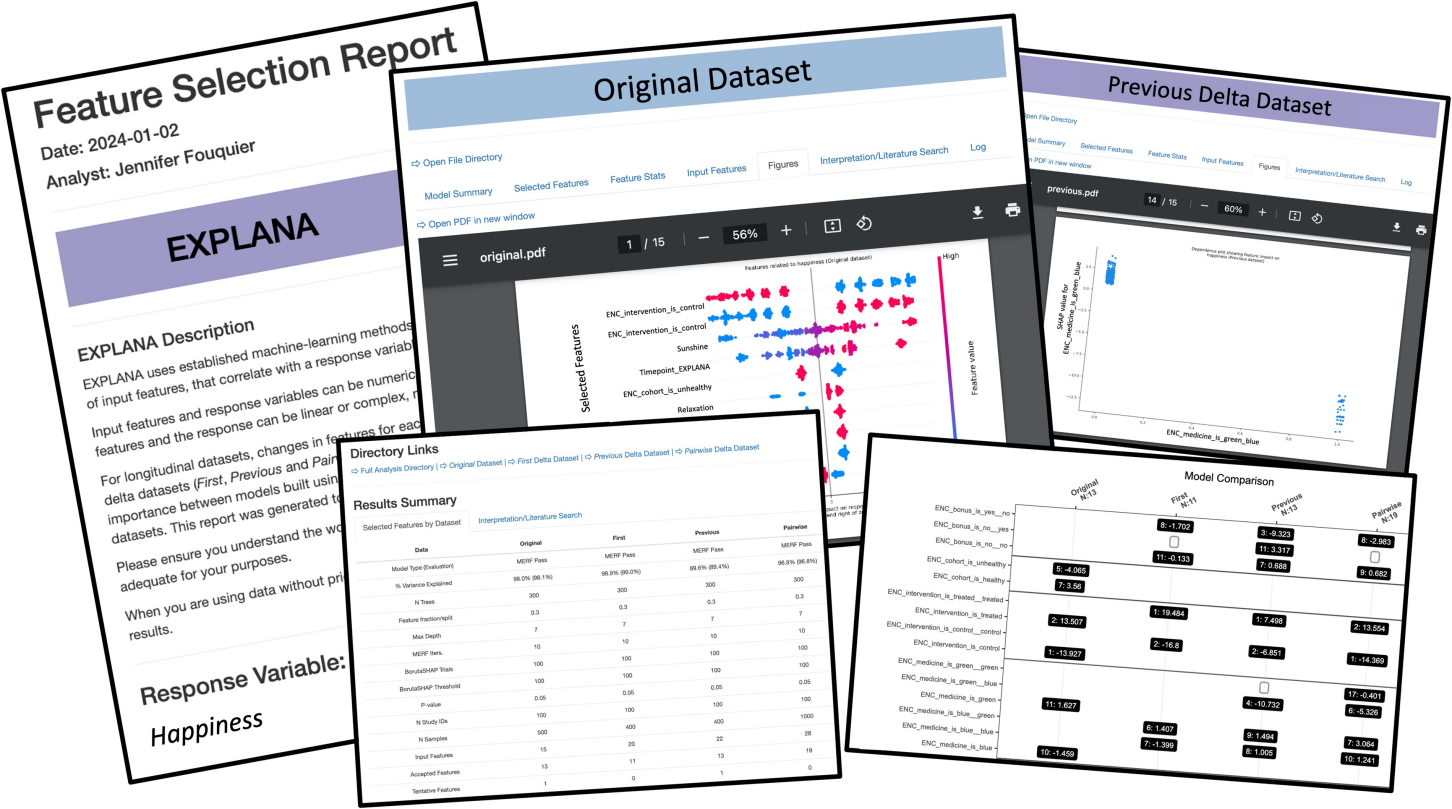
Screenshots from a feature selection report. The report is in an interactive .html format to facilitate interpretation of complex ML method results through figures and written explanations. The report includes a written summary of methods containing arguments and information from the configuration file about the analytic decisions. The dynamic methods can be copy/pasted into manuscripts for efficiency; tables and figures summarize feature selection results for models built from *Original* and, if longitudinal, *First*, *Previous*, and *Pairwise* Δ datasets. Figures include SHAP summary plots and SHAP dependence plots. Links are provided to directories containing files used for report generation to facilitate exploration of data and results.

The result of each analysis is an interactive .html report that includes figures, tables, links to directories for data exploration, links to PubMed for researching findings, and written descriptions of the analytic process (Fig. 3). A methods section is dynamically created based on user inputs or defaults, which can be included in manuscripts. A feature occurrence figure summarizes feature ranks for all models and includes the average impact on outcome (average SHAP value) for positive instances of categorical variables [numerical feature relationships are often more complex (e.g. a hockey curve pattern), so the average SHAP value was not included]. Information about model inputs, selected features, importance scores, and figures, such as SHAP plots, can be found in each of the report sections.

### 2.9 Simulation study design

A simulation study (mock/demo dataset) on happiness was designed to facilitate performance testing using variables that are either predictive or not predictive of happiness. Variables and their effects on the outcome are in Table 1. All individuals started with the same happiness score and effects from all features were used to update each subject’s happiness score at each timepoint. Predictive features had effect values stored in a different column labeled with the suffix “_effect.” For example, the “salary” column contained values “high” or “low” and had a corresponding “salary_effect” column with numerical values reflecting effects on happiness. All effect column values were added to original happiness scores and columns labeled “effect” were removed before feature selection. This way engineered effects were contained in the happiness value, and predictive features can be identified if they corresponded to the effect.

**Table 1.** Performance results from classification accuracy of engineered predictive and not predictive features in five simulation studies.

					BorutaSHAP results			Confusion matrix				Performance results				
Dataset	Software tool	Model	% Variance explained	Input features	Accepted features	Tentative features	Rejected features	True positives	True negatives	False positives	False negatives	Recall (sensitivity)	Precision	Specificity	F1-score	Balanced accuracy
SimFeatures (other input variables; no microbiome)	EXPLANA	Original	98.0% (98.1%)	15	13	1	1	13	2	0	0	1.00	1.00	1.00	1.00	1.00
		First	98.9% (99.0%)	20	11	0	9	10	8	1	1	0.91	0.91	0.89	0.91	0.90
		Previous	89.6% (89.4%)	22	13	1	8	10	8	3	1	0.91	0.77	0.73	0.83	0.82
		Pairwise	96.8% (96.8%)	28	19	0	9	16	7	3	2	0.89	0.84	0.70	0.86	0.79
SimMicrobiome (microbiome; no other input variables)	EXPLANA	*Original*	96.9% (97.0%)	200	28	3	169	23	170	5	2	0.92	0.82	0.97	0.87	0.95
		*First*	96.2% (96.4%)	200	30	3	167	24	169	6	1	0.96	0.80	0.97	0.87	0.96
		*Previous*	65.4% (66.6%)	200	16	1	183	15	174	1	10	0.60	0.94	0.99	0.73	0.80
		*Pairwise*	92.9% (93.1%)	200	40	5	155	25	160	15	0	1.00	0.62	0.91	0.77	0.96
	Q2-longitudinal feature-volatility	*Original*	76.9% (*R*^2^)	200	180	0	20	25	20	155	0	1.00	0.14	0.11	0.24	0.56
SimFeaturesMicrobiome0 (microbiome; other input variables; 0 random variables)	EXPLANA	*Original*	97.4% (97.2%)	215	37	3	175	34	174	3	4	0.89	0.92	0.98	0.91	0.94
		*First*	97.6% (97.7%)	220	32	4	184	31	183	1	5	0.86	0.97	0.99	0.91	0.93
		*Previous*	86.4% (87.9%)	222	32	4	186	28	182	4	8	0.78	0.88	0.98	0.82	0.88
		*Pairwise*	95.5% (95.8%)	228	49	4	175	36	172	13	7	0.84	0.73	0.93	0.78	0.88
SimFeaturesMicrobiome500 (microbiome; other input variables; 500 random variables)	EXPLANA	*Original*	97.3% (97.3%)	715	37	2	676	34	674	3	4	0.89	0.92	1.00	0.91	0.95
		*First*	97.4% (97.7%)	720	32	2	686	30	682	2	6	0.83	0.94	1.00	0.88	0.92
		*Previous*	84.6% (88.3%)	722	37	6	679	27	676	10	9	0.75	0.73	0.99	0.74	0.87
		*Pairwise*	95.1% (95.7%)	728	62	3	663	36	659	26	7	0.84	0.58	0.96	0.69	0.90
SimFeaturesMicrobiome1000 (microbiome; other input variables; 1000 random variables)	EXPLANA	*Original*	97.5% (97.2%)	1215	36	1	1178	34	1175	2	4	0.89	0.94	1.00	0.92	0.95
		*First*	97.4% (97.8%)	1220	38	0	1182	32	1178	6	4	0.89	0.84	0.99	0.86	0.94
		*Previous*	83.9% (87.6%)	1222	45	7	1170	29	1170	16	7	0.81	0.64	0.99	0.72	0.90
		*Pairwise*	95.1% (95.9%)	1228	66	4	1158	36	1155	30	7	0.84	0.55	0.97	0.66	0.91

R package faux (DeBruine 2023; https://doi.org/10.5281/ZENODO.2669586) was used to add subject random effects, five timepoints, and a control and test group. The test group was simulated to linearly increase with a positive slope of 30 (correlation coefficient of 0.7 and SD = 5) to simulate a treatment effect that improved happiness.

To create longitudinal microbiome simulations containing differentially abundant and not differentially abundant microbes, microbiomeDASim (Williams *et al.* 2019) was used with a first-order autoregressive correlation structure that linearly increased with slope 30 to correlate with happiness (correlation coefficient = 0.7; standard deviation = 5). Simulated microbiome data was CLR-transformed prior to creating the *Original* dataset for use in the workflow.

Eight data distributions were used with random, not predictive variables: Normal, Bernoulli, Binomial, Poisson, Exponential, Gamma, Weibull (Korstanje 2025; https://medium.com/data-science/7-statistical-distributions-that-every-data-scientist-should-know-with-intuitive-explanations-bf967db81f0b), and Dirichlet. The number of random variables is indicated in the dataset name. Accordingly, for *SimFeaturesMicrobiome0*, *SimFeaturesMicrobiome500*, and *SimFeaturesMicrobiome1000*, the number of random variables is 0, 500, and 1000, respectively.

When using EXPLANA, arguments were set based on recommendations of the underlying tools or from previous studies on hyperparameter tuning (Probst *et al.* 2019, Weerts *et al.* 2020), which included for MERF, 300 trees, 0.3 feature fraction for decision tree splits with a max depth of 7 and 10 MERF iterations and 100 BorutaSHAP trials were run (100% importance threshold; *P *= .05).

EXPLANA arguments: 300 trees, 0.3 feature fraction for decision tree splits with max depth of 7, 10 MERF iterations, and 100 BorutaSHAP trials at 100% importance score threshold (*P* = .05). Feature-volatility arguments: 300 trees, 5 cross-fold validation. True positives are selected predictive features, true negatives are rejected not-predictive features, false positives are selected not-predictive features, and false negatives are rejected predictive features (Fig. 2, available as supplementary data at *Bioinformatics* online).

### 2.10 Performance evaluation

Performance was assessed using simulation studies and F1-scores and balanced accuracy metrics (Fig. 2, available as supplementary data at *Bioinformatics* online). Recall (TP rate): proportion of predictive features correctly selected (Recall = TP/(TP+FN)). Precision: proportion of all selected features that are truly predictive (Precision = TP/(TP+FP)). An F1-score is calculated using precision and recall 2×(Precision×Recall)/(Precision + Recall), as well as balanced accuracy, which is an average of the sensitivity and specificity.

### 2.11 Early Childhood Antibiotics and Microbiome dataset analysis

To explore feature selection results using published data, feature counts for 455 genera from the Early Childhood Antibiotics and Microbiome (ECAM) dataset were used because the dataset was also used to demonstrate Q2 (Bolyen *et al.* 2019) longitudinal FV (Bokulich *et al.* 2018) functionality. Metadata was filtered to remove duplicate months to facilitate Δ calculations performed by EXPLANA. Five hundred trees were used for both tools. EXPLANA arguments: Feature fraction of 0.3, max depth = 7, with 10 MERF iterations and 100 BorutaSHAP trials (100% threshold; *P* = .05). Q2 FV arguments: Feature fraction of 1.0 (cannot modify) and 5 k-fold cross-validations.

## 3 Results

### 3.1 Software workflow summary

EXPLANA was developed to create a comprehensive feature selection report. The workflow is guided by directions from a configuration file where the user provides dataset paths, selects optional preprocessing steps per dataset, and defines inputs and the outcome of interest (Fig. 1). The input datasets are merged to form the comprehensive *Original* dataset. If more than one timepoint is sampled per subject, feature changes are calculated using different reference points to uncover important features in different contexts of change. Thus, the *Original* dataset is used to compute three Δ datasets, *First*, *Previous*, and *Pairwise* (Fig. 2). Differences are calculated as follows: For *First*, compared to baseline/first measures. For *Previous*, compared to the immediately previous timepoint. For *Pairwise*, all pairwise comparisons between timepoints. Notation throughout is as follows: Timepoints 1, 2, 3, etc. are referred to as T1, T2, T3, etc., respectively. Accordingly, the difference between T1 and T2 is T1_T2, and labeled in chronological order. For T1_T2, T1 is the reference and is subtracted from T2 (Fig. 2).

EXPLANA accommodates numerical and categorical predictor and outcome variables, including novel functionality to track categorical feature changes over time and relate changes to changes in outcome. Microbiome-specific challenges addressed include the option to use a CLR transformation for compositional data (Gloor *et al.* 2017). Additionally, distance matrices such as UniFrac (Lozupone *et al.* 2011) or other beta diversity measures commonly used in microbiome research to evaluate community differences between all samples, can be incorporated during Δ calculations allowing users to evaluate community differences between samples on a per subject basis. Thus, a distance matrix for all samples is subset to become an engineered feature of distances representing the degree of change in an individual’s microbiome community over time. For feature selection, MERF is used as the ML method for repeated measures data, otherwise RF is used. BorutaSHAP (Boruta combined with SHAP) is used to rank features, assess significance, and estimate feature relationship to the outcome.

Specifically, Boruta evaluates whether each input feature is informative of outcome values by comparing it to randomly shuffled versions of all other input features. By repeating this process many times, Boruta gathers statistical evidence to determine feature relevance. While Boruta estimates significance, SHAP is a feature scoring metric that explains how features influence the outcome prediction, considering their impact in combination with other variables. SHAP can indicate if a feature has a positive or negative effect on outcome values or has complex feature interactions. This is an improvement upon conventional feature importance measures, such as Gini or permutation importance, which only rank variables based on their contribution to model predictions. SHAP importance greatly improves interpretability needed for hypothesis generation.

Upon workflow completion, a report is generated that contains a description of the analyses and tables and figures indicating which features were selected (Fig. 3). The ease of running different datasets with EXPLANA enables easier exploratory analyses of datasets, such as performing stratified analyses. For example, to do stratified analyses, the user simply needs to use small R scripts inside the configuration file [e.g. to stratify the dataset by sex, users could include the script df <- df %>% filter(sex == “female”)] which will be documented in the report.

### 3.2 Workflow evaluation and feature selection using a longitudinal simulation study

The workflow was evaluated using longitudinal simulation studies and published datasets (both detailed in Section 2). EXPLANA supports cross-sectional data, although performance was only evaluated using a simulated longitudinal study. A simulated/mock study on longitudinal happiness, called *SimFeatures*, was created for performance evaluation and modeled as an intervention including 100 individuals treated with one of two therapies to improve happiness over five timepoints. Happiness is based on a numerical score where higher values indicate better mental health. For interpretability, features are recognizable as factors that could affect real-life happiness such as relaxation, sunshine, salary, medication, etc. Categorical and numerical features were included with and without relationships to happiness, thus representing predictive (related to outcome) and not predictive (not related to outcome) engineered features (Table 1, available as supplementary data at *Bioinformatics* online). Some features were designed to be important only in some of the four models (Fig. 2; *Original, First, Previous*, or *Pairwise*) to validate whether the tool could select unique features dependent on different contexts of change.

A simulated microbiome feature table was also created using MicrobiomeDASim (Williams *et al.* 2019) with 25 differentially abundant microbes linearly correlated to happiness changes over time and 175 not related to the outcome, which was converted to compositional/proportional data. The dataset with the outcome variable and simulated microbes was called *SimMicrobiome*. The dataset with *SimFeatures* and *SimMicrobiome* combined was called *SimFeaturesMicrobiome0.* Since microbiome datasets can often contain hundreds to thousands of different features, we also evaluated the effects of including many features without a relationship to the outcome. We did this by adding to the *SimFeaturesMicrobiome* dataset an increasing number of random variables from a variety of data distributions. The number of random variables is indicated in the dataset names. Thus, the five simulation studies used for workflow evaluation are *SimFeatures*, *SimMicrobiome*, *SimFeaturesMicrobiome0*, *SimFeaturesMicrobiome500*, and *SimFeaturesMicrobiome1000* (Table 1, available as supplementary data at *Bioinformatics* online; details in Section 2).

Workflow evaluation was performed by appropriate selection or rejection of engineered features. True positives (*selected* predictive features; TPs) and true negatives (*rejected* not-predictive features; TNs) were considered correctly classified, while false positives (*selected* not-predictive features; FPs) and false negatives (*rejected* predictive features; FNs) were considered incorrectly classified (Fig. 2, available as supplementary data at *Bioinformatics* online). These datasets allowed us to: (i) evaluate workflow performance from classification accuracy of engineered features and (ii) to test the hypothesis that unique features dependent on different contexts of change could be identified, including novel order-dependent categorical changes related to an outcome.

**Figure 4.**
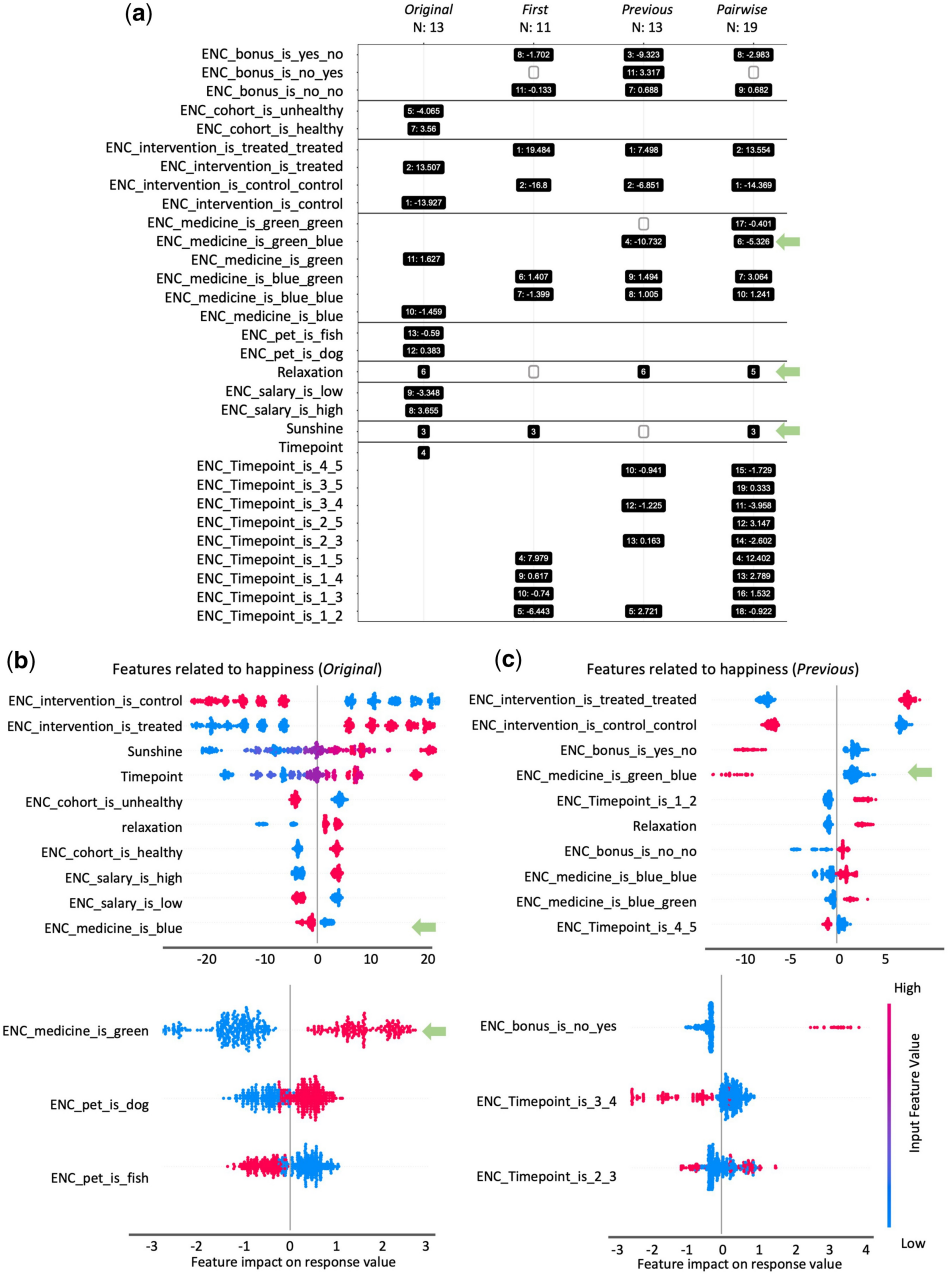
Feature selection report excerpts for one analysis using *SimFeatures* dataset. The *SimFeatures* dataset is a simulated longitudinal intervention with 100 individuals sampled over five timepoints (see Section 2 for detailed description). (a) Feature occurrence figure with selected features organized by model (*Original*, *First*, *Previous*, and *Pairwise*) and ranked with one being the highest importance. For presence (yes/1) of a categorical variable, the average SHAP value is indicated following the colon. This value is not indicated for numerical variables (see Section 2). SHAP summary beeswarm plots for ranked selected features are shown for (b) *Original* and (c) *Previous* models. Each point represents one sample, and the horizontal position indicates impact on the outcome as indicated on the *x*-axis. Points to the left indicate a negative impact, and points to the right indicate a positive impact. The colors represent the selected feature values, where red is larger, and blue is smaller. For binary encoded features (“ENC”) red is yes/1 and blue is no/0. Note that scales differ between the top and bottom SHAP plots, as they are grouped by a maximum of 10 features per SHAP plot. Some features were designed to be identified in only certain models. Green arrows draw attention to key findings explained in Section 3. Interesting results include: ENC_medicine_is_green_blue (pill color), a categorical feature important in *Previous* and *Pairwise* models; “relaxation,” a numerical feature important in *Previous* and *Pairwise* and undetectable in *First* as described in *Discussion*; and “sunshine” which was unable to be detected in *Previous* but a high rank in other models. Multiple analyses create a more comprehensive picture for longitudinal studies. Three hundred were used, with a feature fraction of 0.3, max depth of 7, with 10 iterations of MERFs, and 100 BorutaSHAP trials (100% importance threshold; *P* = .05).

EXPLANA was used to select and rank features related to happiness for the five longitudinal simulation studies for all models (*Original, First, Previous*, or *Pairwise*) to evaluate performance (Fig. 4, Table 1;  Fig. 3, available as supplementary data at *Bioinformatics* online). Balanced accuracy and F1-score (a metric that accounts for both precision and recall; Fig. 2, available as supplementary data at *Bioinformatics* online) respective ranges for *Original, First, Previous*, and *Pairwise* were as follows: 0.79–1.00 and 0.83–1.00 for *SimFeatures*; 0.80–0.96 and 0.73–0.87 for *SimMicrobiome*; 0.88–0.94 and 0.78–0.91 for *SimFeaturesMicrobiome0*; 0.87–0.95 and 0.69–0.91 for *SimFeaturesMicrobiome500*; and 0.90–0.95 and 0.66–0.92 for *SimFeaturesMicrobiome1000. Original* yielded the highest F1-scores and balanced accuracy. The average workflow ability to recall predictive features was good/excellent (average 0.87, SD = 0.09) and good for precision (avg 0.82, SD = 0.14), with some Δ datasets having lower precision or recall. Of the four models analyzed with EXPLANA for *SimMicrobiome*, *Previous* had the lowest percent variation explained (65.4%), a low recall (0.60), and failed to correctly classify some predictive features (Table 1). For simulated datasets with not-predictive microbes or random variables, *Pairwise* had the poorest precision. The lowest F1-score was for *SimFeaturesMicrobiome1000* using *Pairwise* (0.66), which had 30 FPs out of 1000 random variables that affected precision (0.55). However, the proportion of selected predictive features (recall) was good (0.81), and the balanced accuracy was 0.91 (see confusion matrix in Table 1). Results evaluated for all simulated datasets were from one analysis per dataset to ensure consistency with final report, and consistency across 100 runs was assessed using the *SimFeaturesMicrobiome* dataset (Table 2, available as supplementary data at *Bioinformatics* online). Standard deviation for recall, precision, F1-score, balanced accuracy, and percent variation explained was low for *Original*, *First*, *Previous*, and *Pairwise* models (range 0–0.24 SD, with a 0.24 SD for percent variation explained using the *Pairwise* model).

The features selected from the smallest dataset containing a variety of input feature types, *SimFeatures*, are shown in Fig. 4a. Details about features and their motivation for workflow demonstration are explained in Table 1, available as supplementary data at *Bioinformatics* online. One feature that emphasizes the advantage of calculating Δs using the previous values or pairwise values rather than only first/baseline values is pill color, a categorical feature engineered to have a negative impact on happiness when blue pills were taken *after* green (“green_blue”), and not conversely. The feature green_blue had relatively high ranks and a negative impact on the outcome using *Pairwise* and *Previous* (respective rank: impact: *Pairwise *= 6/19: −5.3, and *Previous* 4/13: −10.7; Fig. 4a and c). Green was not a possible value at baseline/T1, therefore green_blue could not be identified with *First*. Green alone was selected with *Original* at a lower importance rank and a small positive impact (rank and impact: 11/13; +1.6) and blue was selected with a small negative impact (rank and impact: 10/13; −1.5). Green occurred at later timepoints, while happiness was also increasing, so selection of green with a small positive impact on happiness was appropriate in the *Original* despite being designed without independent effects. However, without the additional information provided by Δs, an assumption about positive impact on happiness when consuming green pills could have been made.

A numerical example that emphasizes the benefit of using different methods for calculating Δs in longitudinal analysis is “relaxation,” which was selected in *Original*, *Previous*, and *Pairwise* models but not *First*. Relaxation had one value for baseline/T1 and a different value that remained constant at all later timepoints, resulting in T1 comparisons to T2, T3, T4, and T5 having identical values (e.g. T1 = 1, T2–T5 = 5; differences compared to baseline would be 4 in *First*). The lack of change in *First* makes it ineffective for discrimination and pattern recognition for “relaxation” despite its relationship to happiness. Another numerical feature only important in some contexts of change is “sunshine,” which was selected using *Original, First*, and *Pairwise*, but not *Previous*. Sunshine has a linear relationship to happiness over time which can sometimes be less impactful upon calculating Δs using differences between adjacent timepoints only.

EXPLANA can be used to understand beta diversity or community changes over time using distance matrices. This was demonstrated using *SimFeatures* and an Aitchison distance matrix created from *SimMicrobiome*. The distances between samples, per subject, are incorporated into the Δ datasets in the workflow (Fig. 4a, available as supplementary data at *Bioinformatics* online). Aitchison distance was selected for all three Δ datasets and ranked third of 10 (*First*), fifth of 12 (*Previous*), and fourth of 19 (*Pairwise*) selected features (Fig. 4b, available as supplementary data at *Bioinformatics* online).

**Figure 5.**
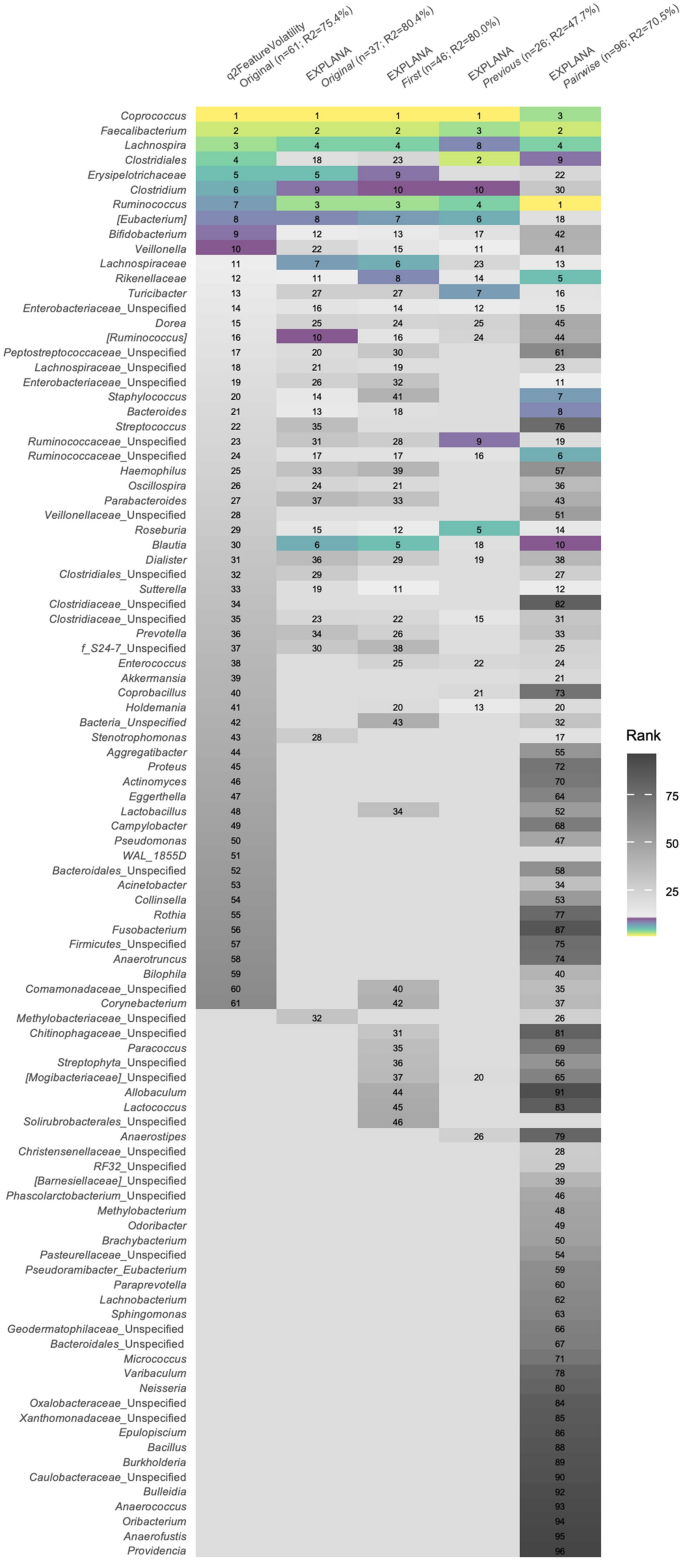
Bacterial genera related to month-of-life in newborns from the ECAM dataset selected using EXPLANA and QIIME 2 longitudinal FV (Bokulich *et al.* 2016, 2018, Bolyen *et al.* 2019). Features are ranked with one being the most important. FV results are in the first column (*Original* model), followed by EXPLANA for *Original*, *First*, *Previous*, and *Pairwise*. Software tool, model and percent variation explained using *R*^2^ (out-of-bag scores from the complete forest) is shown on the *x*-axis. Both tools used 500 trees. EXPLANA had a feature fraction of 0.3, max depth of 7, 10 iterations of MERFs, and 100 BorutaSHAP trials (100% threshold; *P* = .05). For FV, the feature fraction is a fixed parameter at 1.0 and uses RF without mixed effects. Top 10 features per model are emphasized using a sequential multihue color palette from light to dark, and features after 10 are in grayscale from light to dark. *Paracoccus*, *Allobaculum*, *Anaerostipes*, and *Lactococcus* genera were uniquely identified with EXPLANA using Δ datasets. *Blautia* had a much higher importance rank with EXPLANA compared to FV.

EXPLANA can be used with cross-sectional data, which does not include Δs. Performance for cross-sectional data was not evaluated, but was illustrated with *SimFeatures* at timepoint 1. For clarity and ease of interpretation, microbiome data was excluded here. Perfect precision and recall were obtained, with six features selected: high and low salary, fish and dog as pets, healthy and unhealthy individuals (Fig. 5, available as supplementary data at *Bioinformatics* online). Engineered factors with a positive impact on simulated happiness were owning a dog, good health, and high salary.

EXPLANA performance was then compared to an existing feature selection tool for LMS, called Q2 longitudinal (Bokulich *et al.* 2018) FV. Because FV does not create Δ datasets (although other tools in q2-longitudinal can create Δ datasets) and does not automatically include categorical variables like EXPLANA, *SimMicrobiome* (the simulated microbiome, with no other study input variables) was analyzed using FV and EXPLANA (Table 1). Default analysis for FV is on *Original*, and for EXPLANA on *Original, First, Previous*, and *Pairwise*. All performance measures were substantially better for EXPLANA compared to FV, except for recall, which was 0.92 for EXPLANA *Original* and 1.00 for FV (Table 1). Of the 25 predictive microbes, 23 were selected by EXPLANA and 25 by FV and of the 175 not-predictive microbes, EXPLANA selected 5 FPs, while FV selected 155 FPs (balanced accuracy EXPLANA: 0.95 and FV: 0.56).

### 3.3 Feature selection using a published study

EXPLANA was next applied to identify bacteria related to month-of-life in babies from the ECAM study (Bokulich *et al.* 2016) which was also used to compare results to the Q2 longitudinal FV feature selection tool (Bokulich *et al.* 2018, Bolyen *et al.* 2019). The ECAM study generated 16S rRNA targeted sequencing data from monthly fecal samples collected from 43 babies over their first 2 years. Of 455 genera, 61 were selected by FV *Original*, and for EXPLANA, 37 for *Original*, 46 for *First*, 26 for *Previous*, and 96 for *Pairwise* (Fig. 5). Of the 61 genera identified with FV, 25 were rejected by EXPLANA with *Original*. In total, there were 37 genera unique to EXPLANA analyses, with one identified using *Original*, which is an unidentified genus from the family *Methylobacteriaceae*. Thirty-six unique genera were identified using *First, Previous*, and *Pairwise* Δ datasets including *Paracoccus, Allobaculum, Anaerostipes*, and *Lactococcus*, which were each identified in two of the three Δ datasets.

To further demonstrate the versatility for EXPLANA to work with categorical variables using the ECAM dataset, an additional analysis was performed to identify categorical features related to month-of-life, while excluding the numerical microbiome data. Variables included delivery type (cesarean/vaginal), diet during first three months (breast/formula milk), sex (male/female), and antibiotic exposure (yes/no). Change in antibiotic use from no to yes (n_y, an example of order-dependent categorical change) at later timepoints was positively related to month-of-life, indicating that babies are more likely to have an antibiotic treatment event as they age (Fig. 6, available as supplementary data at *Bioinformatics* online). A final analysis was performed using bacteria and categorical data resulting in a combination of categorical and numerical important features selected, including the categorical variable, antibiotic use (Fig. 7, available as supplementary data at *Bioinformatics* online).

## 4 Discussion

To address challenges with identifying variables related to outcomes of interest, a feature-selection tool for longitudinal data was developed to expedite discovery. By design, EXPLANA functions as an efficient way to implement feature selection on cross-sectional data; however, the primary focus of this paper and performance evaluations were for longitudinal data. RFs and MERFs, which are supervised ML methods, were implemented to identify features that relate to outcome values. For meaningful results, it was essential to use methods that not only identify and rank features important for predicting outcome values, but that enhance interpretability and inspire hypotheses. Thus, Boruta evaluates whether features are more important than expected by chance, and SHAP evaluates how a feature impacts the outcome alongside the impact of other variables. These tools improve confidence that significant features were selected and facilitate hypothesis generation by explaining feature impact on dependent outcome variables.

EXPLANA had good performance with simulated data, as determined by selection or rejection of engineered features. The Δ datasets highlighted that change analyses can produce unique insights compared to *Original* longitudinal data without Δ calculations. Several studies have applied MERFs for feature selection (Haran *et al.* 2021, Zeamer *et al.* 2023) as used in EXPLANA. However, they did not use Δs, which could lead to a loss of valuable insights. A limitation of this analysis is that the scoring script, developed uniquely for the simulated happiness dataset, was not integrated into the pipeline, as it would not be generalizable to other datasets. Thus, results are based on a single run. Scoring reliability would improve with multiple runs to reduce Monte Carlo error.

The four models (*Original, First, Previous*, and *Pairwise*) can have strengths and limitations with feature selection. For example, *Previous* failed to select “sunshine” with *SimFeatures*, which had a known linear relationship to happiness over time, while the other three models selected it with a high importance rank. This finding is related to the observation that *Previous* had a low percent variation explained and recall for *SimMicrobiome* because the simulated predictive microbes and sunshine had a linear trend over time. Other temporal trends include quadratic, hockey stick, etc. (Williams *et al.* 2019). *Previous* can miss predictive features when changes are minimized such as when the reference time is closer and predictor variables linearly relate to the outcome. This contrasts with *First*, where changes would be emphasized compared to baseline. A limitation of *First* and *Pairwise* is that Δs are calculated using overlapping timespans, which are not independent (they are temporally autocorrelated). This is particularly an issue with *Pairwise*, which has more Δs. Thus, it is important to consider a higher chance of FPs, which was indeed observed in simulated data. Therefore, stringent statistical parameters should be considered. For all simulations, *First* and *Original* had the best overall percent variation explained as expected for this study design consisting of engineered features with known changes from a baseline value.

Despite model limitations in particular instances, engineered features emphasized the importance in building models using *Original* and Δ datasets. Pill color change “green_blue” was only able to be found in *Previous* and *Pairwise*, and demonstrated the ability for EXPLANA to use Δ datasets to find order-dependent categorical variables that impact an outcome (also seen with “no_yes” for antibiotics in the ECAM data), which are impossible to detect using *Original*. “Relaxation” is impossible to select in *First*, and was not selected, because it lacked variation compared to baseline values; however, it was selected by all other models. These features provide examples that demonstrate how different models, for different contexts of change, are needed to uncover time-dependent effects. The inclusion of ordered variables allows for modeling statistical interactions with time, which are often difficult to interpret, especially with nonlinear approaches like RFs. By incorporating ordered variables in the automated change calculation step, and providing visualizations in the report, the workflow enhances interpretation of findings from longitudinal studies.

Dissimilarities between data or information included in each of the four models can create complications with interpreting feature selection results, such as dropping samples from Δ datasets due to missing timepoints (e.g. unavailable baseline measures for some subjects would exclude them from *First* Δs, while their inclusion would be possible for other models in one report). Another challenge with interpreting results from multiple models arises from including distance matrices, which can only be included in Δ datasets because they represent changes between samples. Thus, care should be taken with interpreting results obtained by different models within one report.

A total of 43 babies were included in the ECAM data reanalysis, which is a relatively small *n*. Other studies have demonstrated that RFs are useful for feature selection with sample sizes in the range of 25–35 samples (Hassan *et al.* 2013, Luan *et al.* 2020, Ward *et al.* 2021). The higher number of important genera selected using FV is likely due to a lack of statistical testing to reduce FPs as done with BorutaSHAP in EXPLANA. Indeed, many FPs were identified using FV with simulated microbiome data (*SimMicrobiome*) which contained a known amount of predictive and not predictive microbes. For the ECAM dataset, importance ranks differ for many genera between EXPLANA and FV leading to different conclusions about the degree of importance regarding developmental microbiome changes. Notably, the genera *Roseburia*, *Ruminococcus*, and *Blautia* were ranked higher with EXPLANA compared to FV, and *Bifidobacterium* and *Veillonella* ranked lower using all four models (Fig. 5). *Blautia* has been associated with breastfeeding and early life events, and is known for beneficial health properties (Liu *et al.* 2021, Freitas *et al.* 2022). Its ranking shift from 30th with FV compared to 6th with EXPLANA highlights the importance of method selection. There were 37 unique genera found by EXPLANA, and not FV.

The tools first differences and first distances (for distance matrices) from q2 longitudinal (Bokulich *et al.* 2018) can create Δs from continuous data, which can be used with FV. However, creation of Δs is not part of the FV feature-selection process, and comparing results from different models is cumbersome without comparative figures, such as the feature occurrences figure provided with EXPLANA (e.g. Fig. 4a). Additionally, the incorporation of categorical Δs is a novel method not performed by other feature selection tools. The ML model used in FV is RF, which is not designed for repeated measures like MERFs used as needed in EXPLANA. The importance score used in FV is Gini, which is biased when categorical and numerical variables are combined (Strobl *et al.* 2007), while EXPLANA uses SHAP, which works well for this combination of feature types. Additionally, SHAP provides feature impact on the outcome, in addition to rank, as well as statistical testing from BorutaSHAP. Overall, analyzing the ECAM data with the mixed-effects models and statistical testing used in EXPLANA yielded a more statistically meaningful set of bacterial genera and unique genera using Δ datasets.

There is no one-size-fits-all model and it is challenging to understand which parameter adjustments will lead to optimal results. However, tuning of the algorithm can address some issues, especially the number of features available per decision tree split (a parameter that cannot be modified in FV), which is affected by the proportion of meaningful and collinear input variables. For preprocessing, while CLR is a common compositional transformation, a recent study showed that relative abundance outperformed compositionally aware methods for ML applications (Yerke *et al.* 2024). This workflow simplifies the process of designing and comparing models to identify effective choices. RF was implemented for many reasons, such as good performance with microbiome data (Zhou and Gallins 2019, Topçuoğlu *et al.* 2020), small datasets and high-dimensional data. However, in some scenarios, other algorithms may be optimal. Specifically, boosting methods can perform better for imbalanced datasets or multiclass classification (Tanha *et al.* 2020). Interpretation of exploratory analysis should be done with care, and *post hoc* testing should be considered.

The barrier to performing data-driven feature selection has been lessened by EXPLANA. Different applications are possible including focusing analysis on specific timepoints or segments of time within a longer study period, such as during plateau or active time periods. Additionally, stratifying by factors such as sex, geography, disease symptom, or a combination of factors could be worthwhile. EXPLANA also provides the opportunity to investigate different variables as inputs, sets of inputs, or as outcomes from prior hypotheses or from results of another exploratory analysis.

Overall, EXPLANA addresses many challenges of high-dimensional exploratory data analysis by combining existing tools and novel methods and facilitates data-driven hypothesis generation.

## Supplementary Material

btaf658_Supplementary_Data

## Supplementary data

Conflict of interest: J.F. receives compensation from commercial licensing agreements and consulting. Other authors declare no competing interests.
