# Supplementary material for: EXPLANA: a user-friendly workflow for EXPLoratory ANAlysis and feature selection in cross-sectional and longitudinal microbiome studies
Source: Bioinformatics. 2025 Dec 19;42(1):btaf658. doi: 10.1093/bioinformatics/btaf658 (PMC12766912; doi:10.1093/bioinformatics/btaf658)
Supplement: btaf658_Supplementary_Data [file btaf658_supplementary_data.pdf]

Supplemental Figures

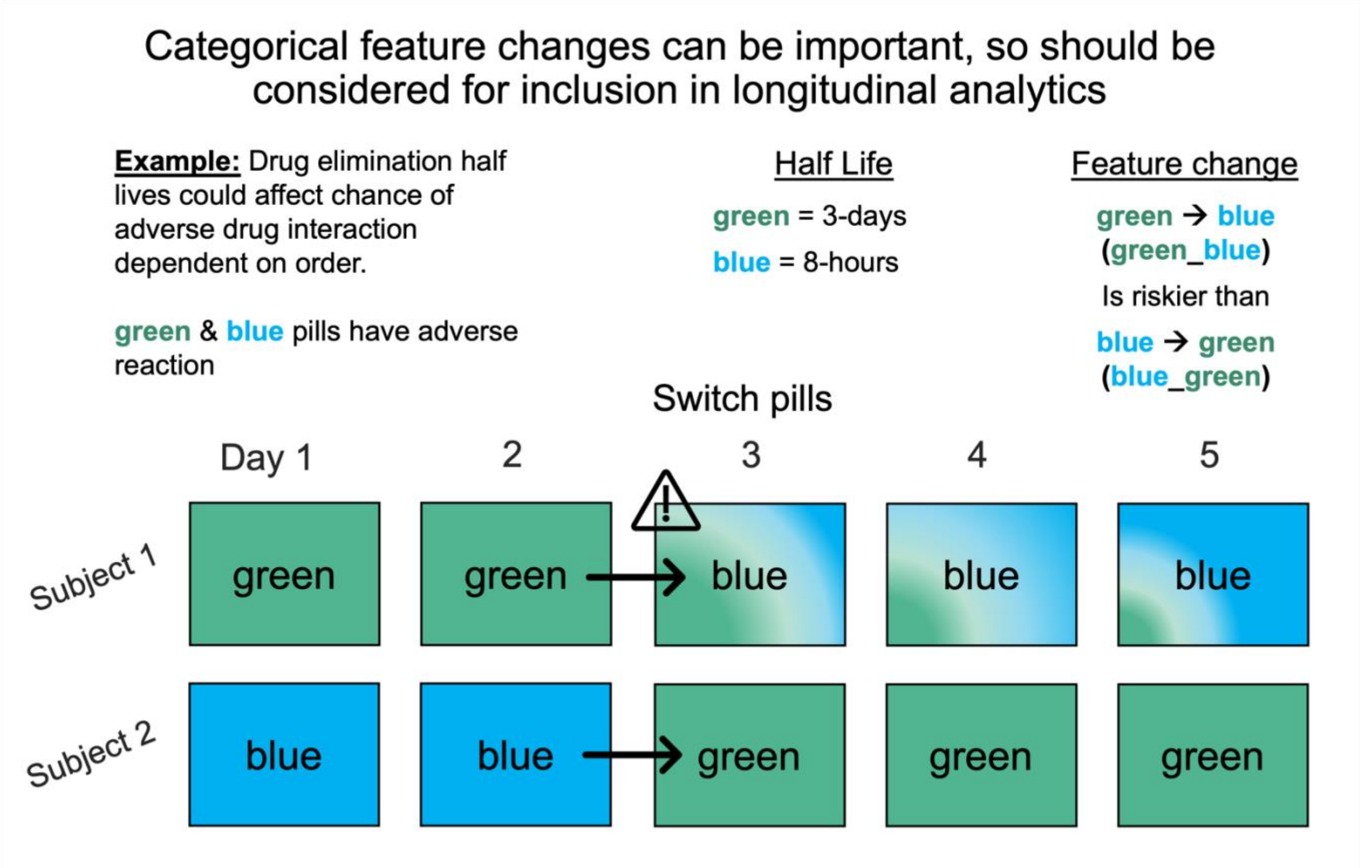

Supplemental Figure 1. Impact of order-dependent categorical feature changes on outcome. One example to demonstrate order-dependent categorical feature changes and differential impact involves two heart arrhythmia treatment medications: a long half-life drug like amiodarone, and a shorter half-life drug like quinidine. Their interaction can cause rapid heartbeat as an adverse effect. This implies that the long half-life drug taken first is riskier. Other examples of categorical feature changes that may have a differential impact on results include lot number changes, weather patterns, supply chain or logistics, patient care, behavior, etc. Incorporating categorical data in longitudinal analytics can lead to novel findings.

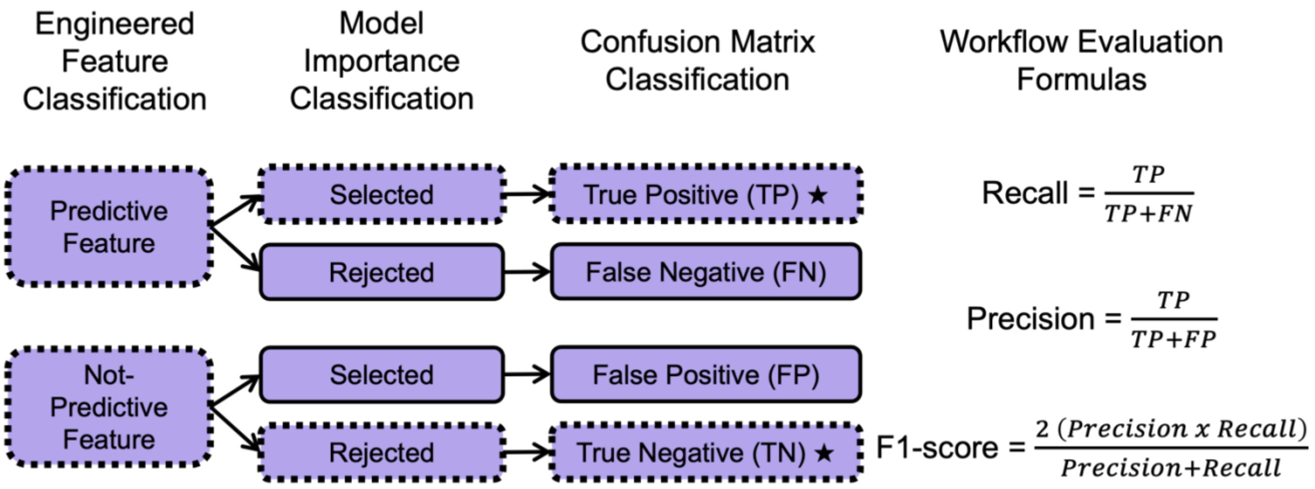

Supplemental Figure 2. Classification diagram for engineered predictive and not predictive features used for model performance evaluation. The simulation study contains features with a relationship to the outcome variable (predictive features) and without (not-predictive features). Dashed lines and stars indicate the correct classification paths for engineered features. Recall (TP rate) is the proportion of predictive features correctly selected (Recall = TP/(TP+FN)). Precision is the proportion of all selected features that are truly predictive (Precision = TP/(TP+FP)). An F1-score is calculated using precision and recall (2*(Precision * Recall) / (Precision + Recall)). TP = true positive, FP = false positive.

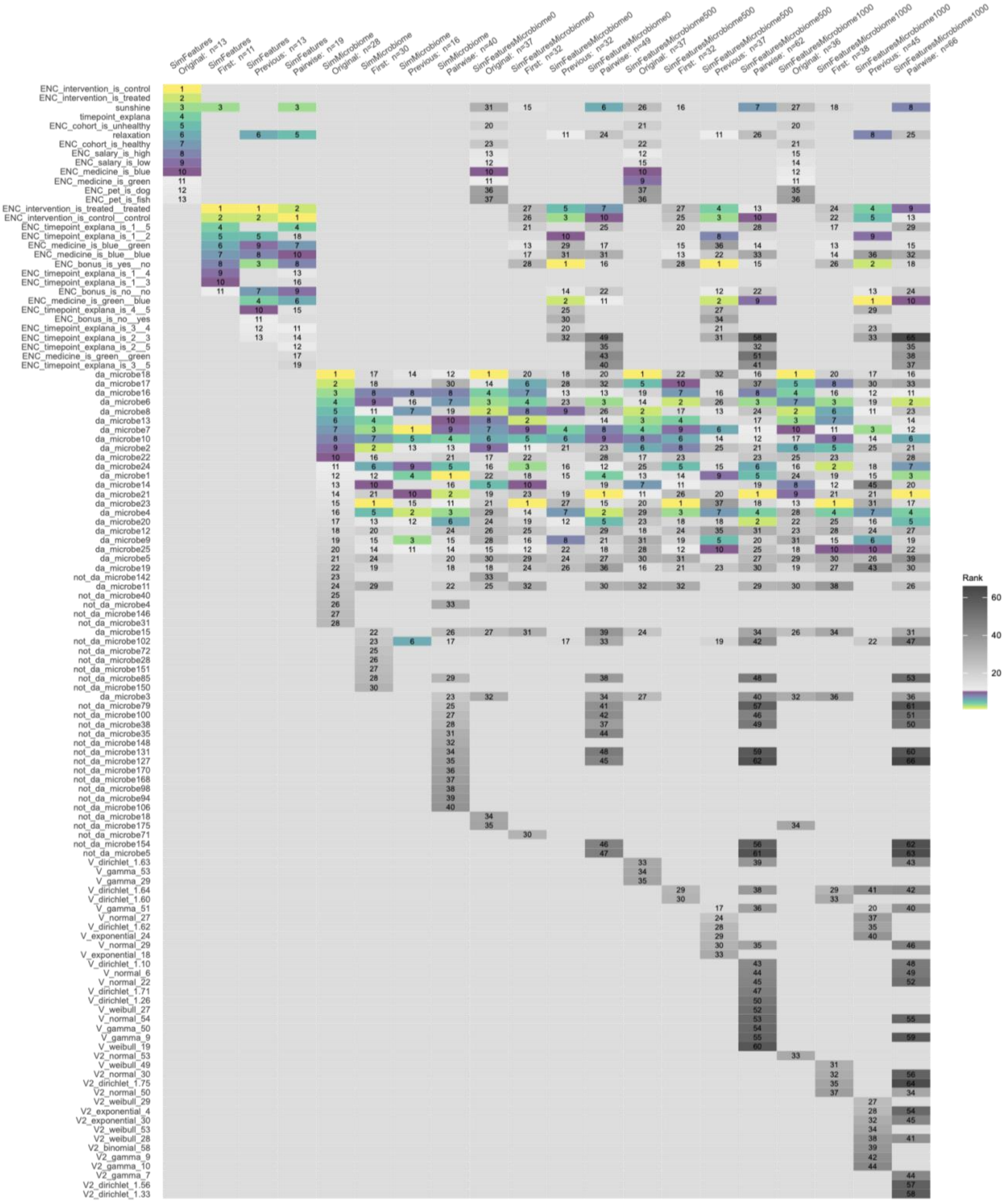
SimFeatures Original: n=13
SimFeatures First: n=11
SimFeatures Previous: n=13
SimFeatures Pairwise: n=19
SimMicrobiome Original: n=28
SimMicrobiome First: n=30
SimMicrobiome Previous: n=16
SimMicrobiome Pairwise: n=40
SimFeaturesMicrobiome0 Original: n=37
SimFeaturesMicrobiome0 First: n=32
SimFeaturesMicrobiome0 Previous: n=32
SimFeaturesMicrobiome0 Pairwise: n=49
SimFeaturesMicrobiome500 Original: n=37
SimFeaturesMicrobiome500 First: n=32
SimFeaturesMicrobiome500 Previous: n=37
SimFeaturesMicrobiome500 Pairwise: n=62
SimFeaturesMicrobiome1000 Original: n=36
SimFeaturesMicrobiome1000 First: n=38
SimFeaturesMicrobiome1000 Previous: n=45
SimFeaturesMicrobiome1000 Pairwise: n=66
Rank
60
40
20

Supplemental Figure 3. Features related to happiness selected using EXPLANA with five variations of simulated longitudinal microbiome datasets. The *SimFeatures* dataset is a simulated longitudinal intervention with 100 individuals sampled over five timepoints. *SimMicrobiome* is a simulated longitudinal microbiome dataset created using MicrobiomeDASim[37] with a ratio of 25 differentially abundant microbes to 175 not differentially abundant. SimMicrobiome was CLR-transformed prior to workflow implementation. See Methods and Supplemental Table 1 for detailed description of the simulation study design. *SimFeaturesMicrobiome0*, *SimFeaturesMicrobiome500*, and *SimFeaturesMicrobiome1000* are dataset variations that include a simulated microbiome, study variables and random variables from a variety of data distributions with no relationship to the outcome. The number in the dataset name represents the number of random variables included. 300 trees were used, with a feature fraction of 0.3, max depth of 7, with 10 iterations of mixed-effects Random Forests (MERFs), and 100 BorutaSHAP trials (100% importance threshold at p=0.05). Top ten features per model are emphasized using a sequential multi-hue color palette from light to dark, and features after 10 are in grayscale from light to dark. Notable features include “sunshine,” which was selected in *Original, First* and *Pairwise*, but not selected in *Previous*; “relaxation” which was not selected in *First* but was selected in *Original, Previous* and *Pairwise*; and “green_blue,” an order-dependent categorical feature that impacted the response and is only able to be found using delta ∆ datasets.

a)

Distance Matrix				
	Subject1_time1	Subject1_time2	Subject2_time1	Subject2_time2
Subject1_time1	0			
Subject1_time2	3	0		
Subject2_time1	6	5	0	
Subject2_time2	10	9	18	0
Distances between time 1 and 2				
	delta_distance_matrix			
Subject1_time1__Subject1_time2	3			
Subject2_time1__Subject2_time2	18			

b)

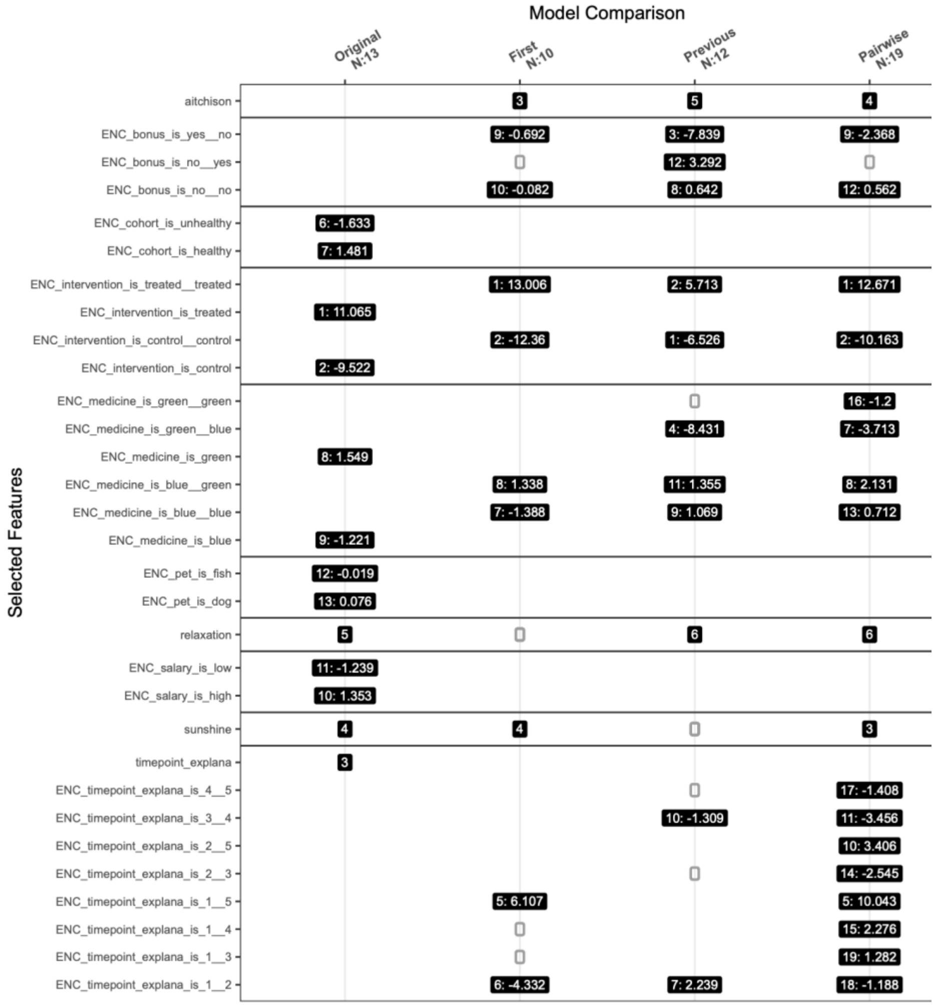

Supplemental Figure 4: EXPLANA feature selection results highlighting the importance of beta diversity changes using distance matrices. The *SimFeatures* dataset was analyzed with an Aitchison distance matrix created from *SimMicrobiome* to identify features associated with simulated happiness. (a) Schematic showing how per-subject distances between samples are incorporated into delta datasets prior to model building. (b) Feature occurrence plot displaying feature ranks and SHAP values. Aitchison distance was selected as important in all three delta datasets, ranking 3rd of 10 (*First*), 5th of 12 (*Previous*), and 4th of 19 (*Pairwise*).

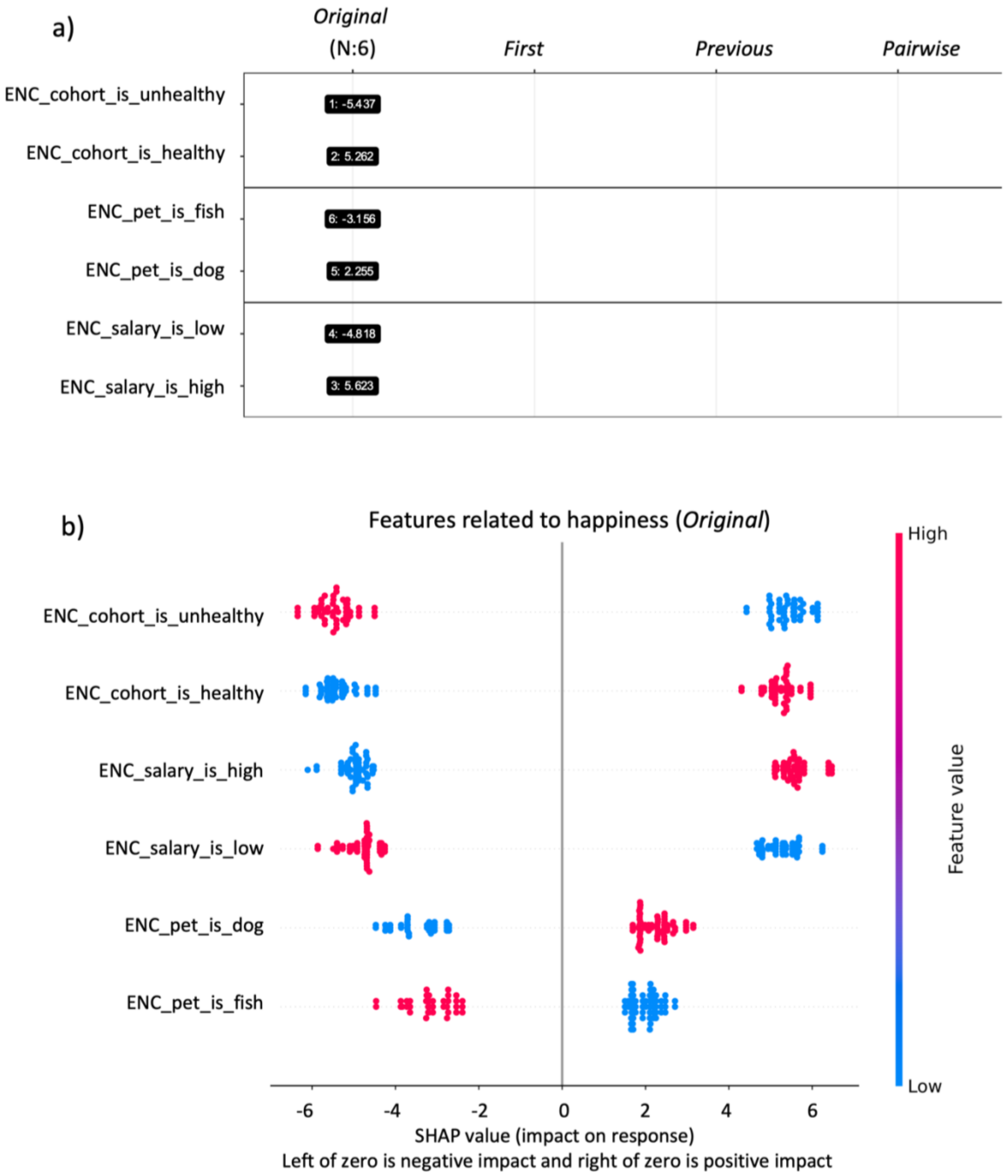

Supplemental Figure 5. Cross-sectional feature-selection results using EXPLANA with baseline values only from simulated happiness dataset. *SimFeatures* dataset was used at timepoint 1 and cohort, salary and pet were selected as important. (a) Feature occurrence diagram displaying rank and SHAP value. Unhealthy individuals are ranked 1 and have a -5.4 impact on “happiness” and healthy individuals are ranked 2 and have a 5.2 impact. (b) SHAP summary beeswarm plot where each point represents one sample, and the horizontal position indicates impact on the outcome as indicated on the x-axis. Points to the left indicate a negative impact, and points to the right indicate a positive impact. The colors represent the selected feature values, where red is larger, and blue is smaller. For binary encoded features (‘ENC’) red is yes/1 and blue is no/0. Features are ordered by largest to smallest impact on the response. As shown, low salary negatively impacts happiness and having a dog positively impacts happiness.

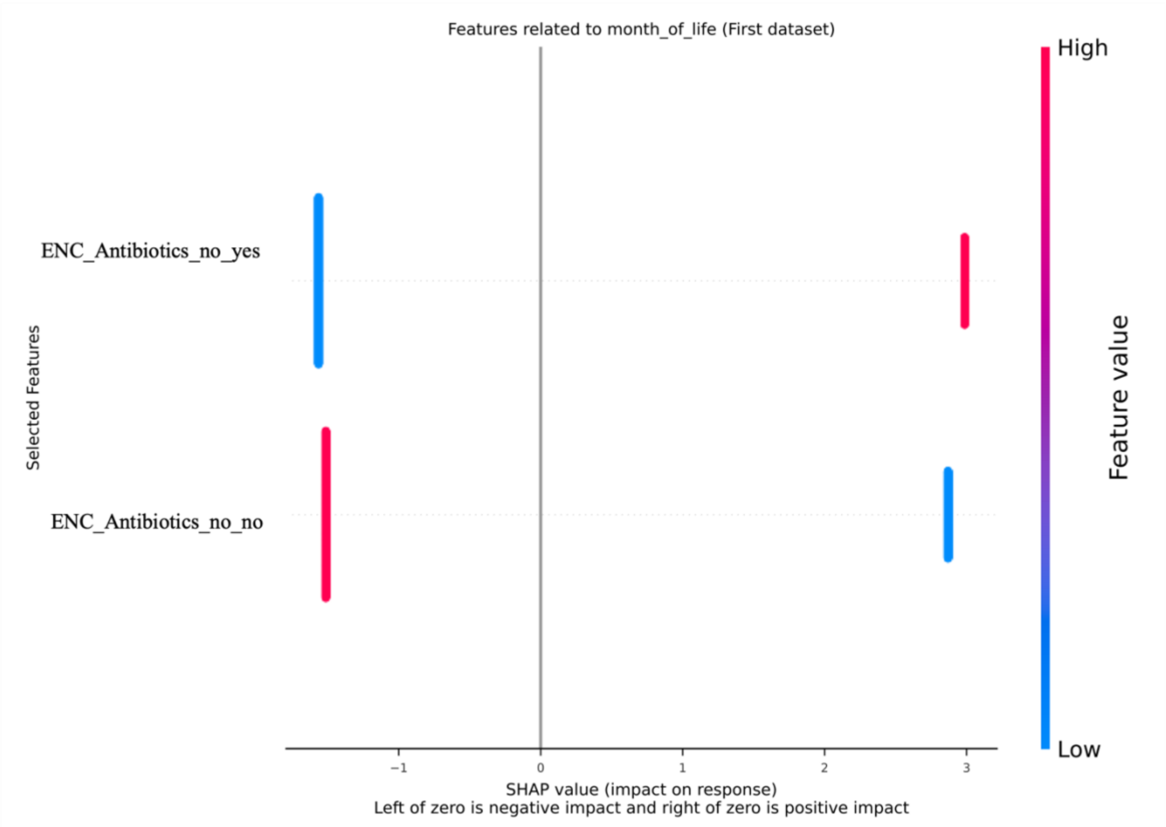

Supplemental Figure 6. Categorical features predictive of month-of-life in newborns selected by EXPLANA using the Early Childhood and Microbiome (ECAM) dataset. RF was used with 500 trees, a feature fraction of 0.2, max depth of 7, 10 MERF iterations, and 100 BorutaSHAP trials (100% threshold; p=0.05). Each point represents one sample, and the horizontal position indicates impact on the outcome as indicated on the x-axis. Points to the left indicate a negative impact, and points to the right indicate a positive impact. The colors represent the selected feature values, where red is larger, and blue is smaller. For binary encoded features ('ENC') red is yes/1 and blue is no/0.

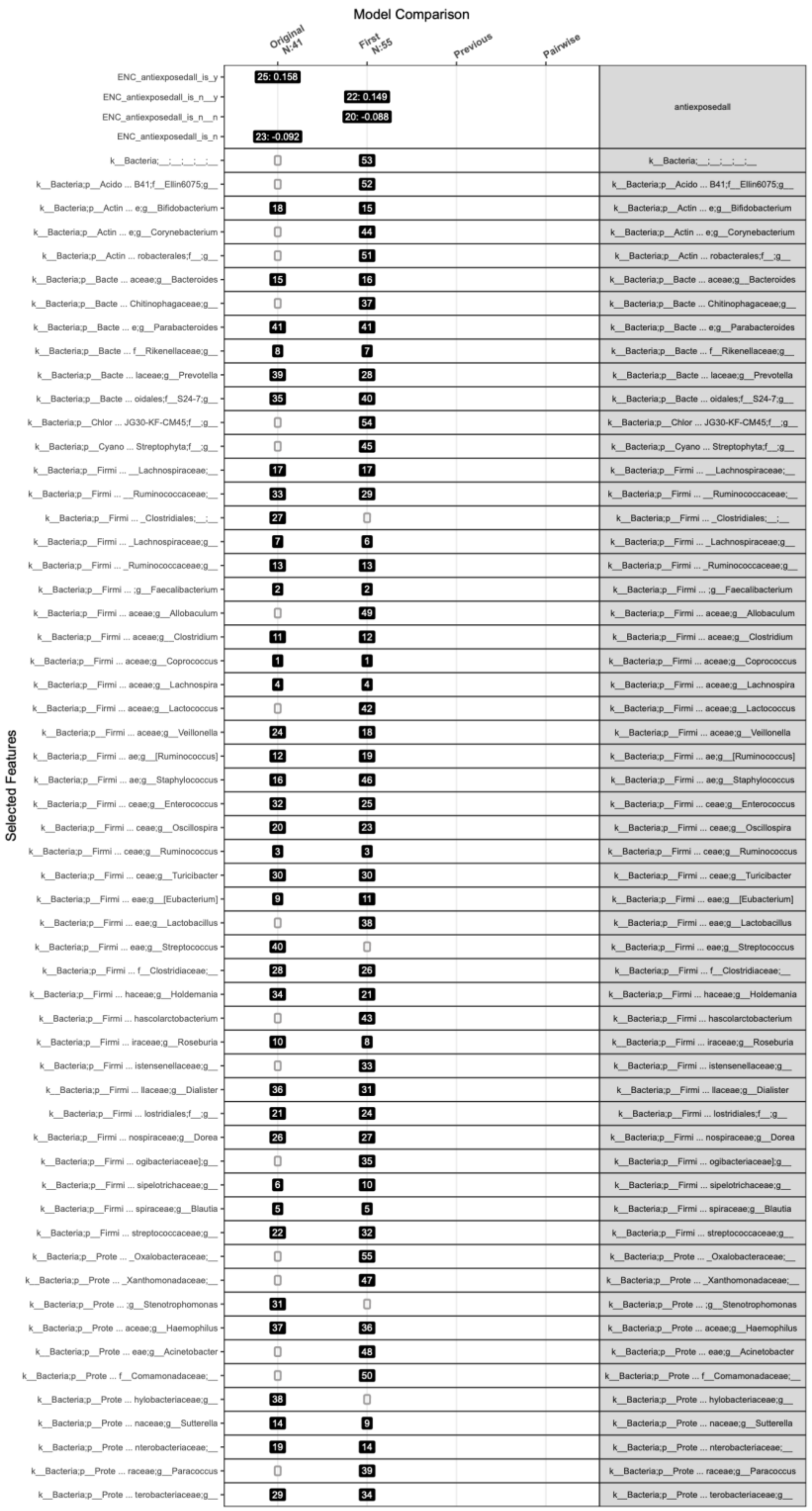

Supplemental Figure 7. Categorical features and bacteria predictive of month-of-life in newborns selected by EXPLANA using the Early Childhood and Microbiome (ECAM) dataset. RF was used with 500 trees, a feature fraction of 0.2, max depth of 7, 10 MERF iterations, and 100 BorutaSHAP trials (100% threshold; p=0.05).

Supplemental Tables

Supplemental Table 1. Engineered predictive (related to outcome) and not-predictive (not related to outcome) input features used in happiness simulation studies.

Input variable	Predictive or Not Predictive	Categorical or Numerical	Motivation for inclusion	Description of expected effect
Timepoint	Predictive	Numerical (original dataset); Categorical (delta datasets)	Timepoint is needed for longitudinal models and helps explain changes over time for other features	Correlation with changes in happiness over time
Cohort	Predictive	Categorical	feature with a positive impact on happiness and no relationship to time	Unhealthy = negative effect; healthy = positive effect
Intervention	Predictive	Categorical	has both a time-independent positive impact on happiness and a time-dependent, positive linear relationship to happiness	Treated and therapy_2: positive linear relationship to time with therapy_1 having a bigger impact
Pill color	Predictive	Categorical	Categorical variable with feature changes that impact response in an order-dependent manner	green_blue has negative impact (can only be identified in Previous and Pairwise models as green did not occur at baseline)
Sunshine	Predictive	Numerical	Numerical variable with positive linear relationship to response	Positive linear relationship
Relaxation	Predictive	Numerical	Effect that plateaus and remains constant (for varying patterns of change over time)	T1 has one value; T2, T3, T4, T5 have the same equivalent value (different than T1)
Salary	Predictive	Categorical	Categorical variable with two features that impact response	High has a positive impact and low has a negative impact
Pet	Predictive	Categorical	Categorical variable with two features that impact response	
Random data distributions: normal, Bernoulli, binomial, Poisson, exponential, gamma, Weibull, and Dirichlet	Not Predictive	Numerical and Categorical representation (binary data distribution)	To test workflow performance with random variables that are not predictive. Random variables are included in *SimFeaturesMicrobiome500* and *SimFeaturesMicrobiome1000*	No anticipated relationships
Differentially abundant microbes	Predictive	Numerical	To test workflow performance with sparse, compositional simulated microbes that increase over time along with response. Included in *SimMicrobiome* and *SimFeaturesMicrobiome0*, *SimFeaturesMicrobiome500,* and *SimFeaturesMicrobiome1000*	Positive linear relationship to happiness
Not differentially abundant microbes	Not Predictive	Numerical	To test workflow performance with compositional data that does not increase over time. Included in *SimMicrobiome* and *SimFeaturesMicrobiome0*, *SimFeaturesMicrobiome500,* and *SimFeaturesMicrobiome1000*	No anticipated relationships

Supplemental Table 2. Performance evaluation over 100 runs using SimFeaturesMicrobiome

	Recall Mean	Recall SD	Recall Min	Recall Max
first	0.86	0	0.86	0.86
original	0.89	1.12E-16	0.89	0.89
previous	0.84	2.23E-16	0.84	0.84
pairwise	0.78	1.12E-16	0.78	0.78

	Precision Mean	Precision SD	Precision Min	Precision Max
first	1	0	1	1
original	0.92	1.12E-16	0.92	0.92
previous	0.72	1.12E-16	0.72	0.72
pairwise	0.88	0	0.88	0.88

	F1-score Mean	F1-score SD	F1-score Min	F1-score Max
first	0.93	1.12E-16	0.93	0.93
original	0.91	2.23E-16	0.91	0.91
previous	0.77	3.35E-16	0.77	0.77
pairwise	0.82	1.12E-16	0.82	0.82

	Balanced Accuracy Mean	Balanced Accuracy SD	Balanced Accuracy Min	Balanced Accuracy Max
first	0.93	1.12E-16	0.93	0.93
original	0.94	3.35E-16	0.94	0.94
previous	0.88	0	0.88	0.88
pairwise	0.88	0	0.88	0.88

	% Variation Explained Mean	% Variation Explained SD	% Variation Explained Min	% Variation Explained Max
first	97.53	0.04819992	97.4	97.6
original	97.377	0.048938221	97.3	97.5
previous	95.436	0.052261966	95.3	95.6
pairwise	86.426	0.23978947	85.9	87

Variation of performance metrics across 100 EXPLANA runs using the SimFeaturesMicrobiome simulated dataset. Each analysis used 300 trees, 10 MERF iterations, 0.3 feature fraction, 100 BorutaSHAP trials, 100% threshold and p = 0.05.
